# 7-Valent Pneumococcal Conjugate Vaccination in England and Wales: Is It Still Beneficial Despite High Levels of Serotype Replacement?

**DOI:** 10.1371/journal.pone.0026190

**Published:** 2011-10-14

**Authors:** Yoon Hong Choi, Mark Jit, Nigel Gay, Nick Andrews, Pauline A. Waight, Alessia Melegaro, Robert George, Elizabeth Miller

**Affiliations:** 1 Immunisation, Hepatitis and Blood Safety Department, Health Protection Agency, London, United Kingdom; 2 Modelling and Economics Unit, Health Protection Agency, London, United Kingdom; 3 Fu Consulting, Hungerford, Berkshire, United Kingdom; 4 Statistics Unit, Health Protection Agency, London, United Kingdom; 5 DONDENA Centre for Research on Social Dynamics, Bocconi University, Milan, Italy; 6 Respiratory and Systemic Infection Laboratory, Health Protection Agency, London, United Kingdom; Health Protection Agency, United Kingdom

## Abstract

**Background:**

The UK introduced the 7-valent pneumococcal conjugate vaccine (PCV7) into the national vaccination program in September 2006. Previous modelling assumed that the likely impact of PCV7 on invasive pneumococcal disease (IPD) would be similar to the US experience with PCV7. However, recent surveillance data show a more rapid replacement of PCV7 IPD cases by non-PCV7 IPD cases than was seen in the US.

**Methods and Findings:**

A previous model of pneumococcal vaccination was re-parameterised using data on vaccine coverage and IPD from England and Wales between 2006 and 2009. Disease incidence was adjusted for the increasing trend in reported IPD cases prior to vaccination. Using this data we estimated that individuals carrying PCV7 serotypes have much higher protection (96%;95% CI 72%-100%) against acquisition of NVT carriage than the 15% previously estimated from US data, which leads to greater replacement. However, even with this level of replacement, the annual number of IPD cases may be 560 (95% CI, -100 to 1230) lower ten years after vaccine introduction compared to what it may have been without vaccination. A particularly marked fall of 39% in children under 15 years by 2015/6 is predicted.

**Conclusion:**

Our model suggests that PCV7 vaccination could result in a decrease in overall invasive pneumococcal disease, particularly in children, even in an environment of rapid replacement with non-PCV7 serotypes within 5 years of vaccine introduction at high coverage.

## Introduction

Pneumococcal disease is a major cause of morbidity and mortality in young children, the elderly and those with underlying chronic conditions such as asplenia and immunosuppression. Polysaccharide vaccines covering 23 of the most prevalent pneumococcal serotypes (PPV23) provide limited protection against invasive pneumococcal disease (IPD) and have not had a major impact on the overall burden of pneumococcal- attributable disease in those targeted for vaccination [1]. Moreover PPV23 does not protect against acquiring carriage of *Streptococcus pneumoniae*, and hence provides no indirect protection in the unvaccinated. Highly immunogenic pneumococcal conjugate vaccines in which the polysaccharide antigens are covalently linked with a protein provide superior protection against IPD and also reduce carriage of vaccine type (VT) organisms thus generating herd immunity against VT disease in unvaccinated groups. Reduction in carriage of VT organisms can however lead to an increase in carriage of non-VT (NVT) organisms – a phenomenon known as serotype replacement [2], [3].

A seven-valent pneumococcal conjugate vaccine (PCV7), providing protection against serotypes 4, 6B, 9V, 14, 18C, 19F and 23F was introduced into the routine childhood immunisation programme in the United States (US) in 2000 and was shown to reduce the incidence of VT IPD in all age groups with little NVT replacement disease [4]. Based on the favourable US experience a number of countries have now introduced PCV7, including the United Kingdom (UK) where since September 2006 PCV7 has been given at 2, 4 and 13 months with an initial catch up immunisation programme up to two years of age. The likely impact of PCV7 in the UK was initially evaluated using a dynamic transmission model with vaccine impact parameterised on the basis of post-PCV7 IPD data from the US. This model predicted elimination of VT IPD with limited serotype replacement such that there was a sustained reduction in overall IPD in all age groups [5]. However, the post-PCV7 experience in England and Wales showed more aggressive serotype replacement than reported in the US with consequently less impact than expected on the overall incidence of IPD [6]. Similar increases in NVT IPD which have largely negated the reduction in VT IPD have been reported elsewhere [7], [8].

Given the different post-PCV7 experience in the UK, we have used the national surveillance data on IPD from the first three years post- PCV7 introduction in England and Wales to re-estimate key parameters for the dynamic transmission model. One of the most important is the competition parameter which reflects the extent to which carriage of one serotype protects against acquisition of another. If there is little protection against pneumococcal co-colonisation when carrying VT organisms then their removal from carriage will have little effect on acquisition and carriage of NVT and little propensity for serotype replacement. Conversely, if the protection against pneumococcal co-colonisation when carrying VT organisms is high then their removal from carriage has the potential to increase the acquisition and carriage of NVT pneumococci, which will subsequently be manifest as replacement disease. The competition parameter is derived from the observed changes in VT and NVT IPD before and after PCV7 introduction. Also, since the UK PCV7 programme, unlike that in the US, rapidly achieved high coverage both in the routine and the catch up programme, we fitted the model using the actual age-specific coverage data for eligible monthly birth cohorts achieved by the programme. We used the re-parameterised dynamic transmission model to predict the long term impact of the PCV7 programme on the incidence of VT and NVT IPD by age in England and Wales.

## Methods

### Model structure

**Figure 1:**
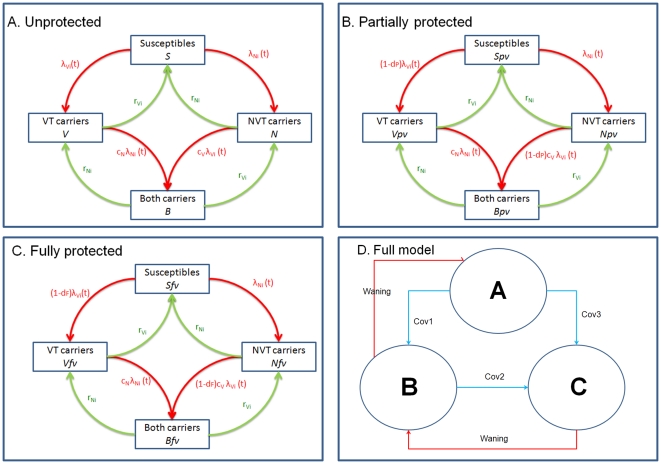
Flow diagrams of the pneumococcal transmission dynamics model structures for pre- and post- PCV7 introduction. Sus: susceptibles, VT: PCV7 type carriers, NVT: non-PCV7 type carriers, Both: both carrying VT and NVT. A) Pre-PCV7 or Unprotected group, B) Partially Protected, C) Fully Protected, and D) Movements between three groups (A, B and C) through vaccination and waning. The r parameters are age-dependent recovery rates, lambdas are forces of infection, and c_N_ and c_V_ are competition parameters. The VEc for Partially and Fully Protected groups are denoted as *d_P_* and *d_F_* respectively.

The pneumococcal transmission model describes the dynamics of carriage of *S. pneumoniae,* with the resultant expression as IPD dependent on the case:carrier ratio.(i.e the propensity to cause disease if carried) which varies between serotypes [9], [10]. The model consists of a set of differential equations that describe the transmission and clearance of carriage (Figure 1A). A Susceptible-Infected-Susceptible (SIS) type model was assumed with no natural immunity to subsequent infection after clearing carriage. Individuals infected by a VT organism could be co-infected by a NVT organism or vice versa. Hence, this model contains four categories of compartments: susceptibles (non-carriers); VT (VT carriers); NVT (NVT carriers); both (carriers of both VT and NVT).

Carriers are able to transmit infection of the same type (VT or NVT) to a susceptible individual at a rate determined by the rate of physical contacts between individuals in six age groups (<2, 2–4, 5–9, 10–19, 20–39, and 40+) in the UK estimated in the POLYMOD diary-based survey of contacts [11], as well as the risk of transmission per contact. Sensitivity analysis conducted on the frequency of contacts used in the model was performed by using all contacts (rather than just physical contacts) from the POLYMOD study. The wider age band of over 40 is necessitated by the small number of positive samples in the carriage data in older individuals [12]; a similar low carriage prevalence was shown in a carriage study conducted among elderly cohorts in Sydney, Australia [13]. The risk of transmission per contact is unknown and was determined by fitting to data (see below). Since the risk may depend on the age of the infected individual, up to 12 parameters may be employed (6 probabilities for six age groups in each PCV7-related serotype groups (VT or NVT)). Four different models were constructed with the following number of parameters governing this risk: (i) two (assuming the same risks for all age groups), (ii) four (assuming children under 2 years old have different risks), (iii) six (assuming stratification into <2, 2-4 and 5+ year olds), (iv) eight (assuming stratification into <2, 2–4, 5–9 and 10+ year olds). Individuals in the population can be unprotected (Figure 1A), partially protected (Figure 1B) or fully protected (Figure 1C) by vaccination. Children who received one dose under the age of one year only get partial protection while those who received at least 2 doses following the first dose under the age of one and those who received at least 1 dose above the age of one year get full protection. Vaccine protection for children who received 2 doses can wane so that the children will belong to either the Unprotected or the Partially Protected compartments. If these children receive the third dose at 13 months, they regain the vaccine protection and become fully protected. This is an improvement over our previous model [5] which only had two vaccine protected states, to allow more realistic representation of actual vaccine uptake in England and Wales. Each month, a proportion of the remaining unprotected or partially protected children move to partially protected or fully protected groups (Figure 1D). Individuals who are partially or fully vaccine protected gradually lose their protection and move to unprotected or partially protected compartments respectively, at a waning rate *w* ( = 1/duration of protection) that is constant throughout all ages and across all compartments. Vaccine protection reduces the probability of acquiring VT carriage (with efficacy VEc), and the risk of developing invasive disease following VT carriage (with efficacy VEd).

The differential equations for the model in Figure 1 are presented in Figure S1.

### Population structure

The population in the model is divided into 100 annual age-cohorts (0, 1, 2, 3, … , 99). Each annual age-cohort is divided into 48 equal-sized age-cohorts (in total 4800 age-cohorts in the total population in the model), for the convenience of having four time steps in each month when using monthly vaccine uptake data. Individuals are born into the first age-cohort 0, and die after the last age-cohort 4800. All 4800 age-cohorts begin with a fixed number of individuals (1000). However, when calculating the force of infection, the proportion of infected individuals in each compartment is weighed by the actual number of people in each age group (based on Office for National Statistics figures from year 2005, i.e. the year immediately prior to the period being modelled). This ensures a more realistic representation of the population structure than alternative methods incorporating a fixed or age-dependent mortality rate (see Figure S2).

### Data sources

#### IPD incidence

**Figure 2:**
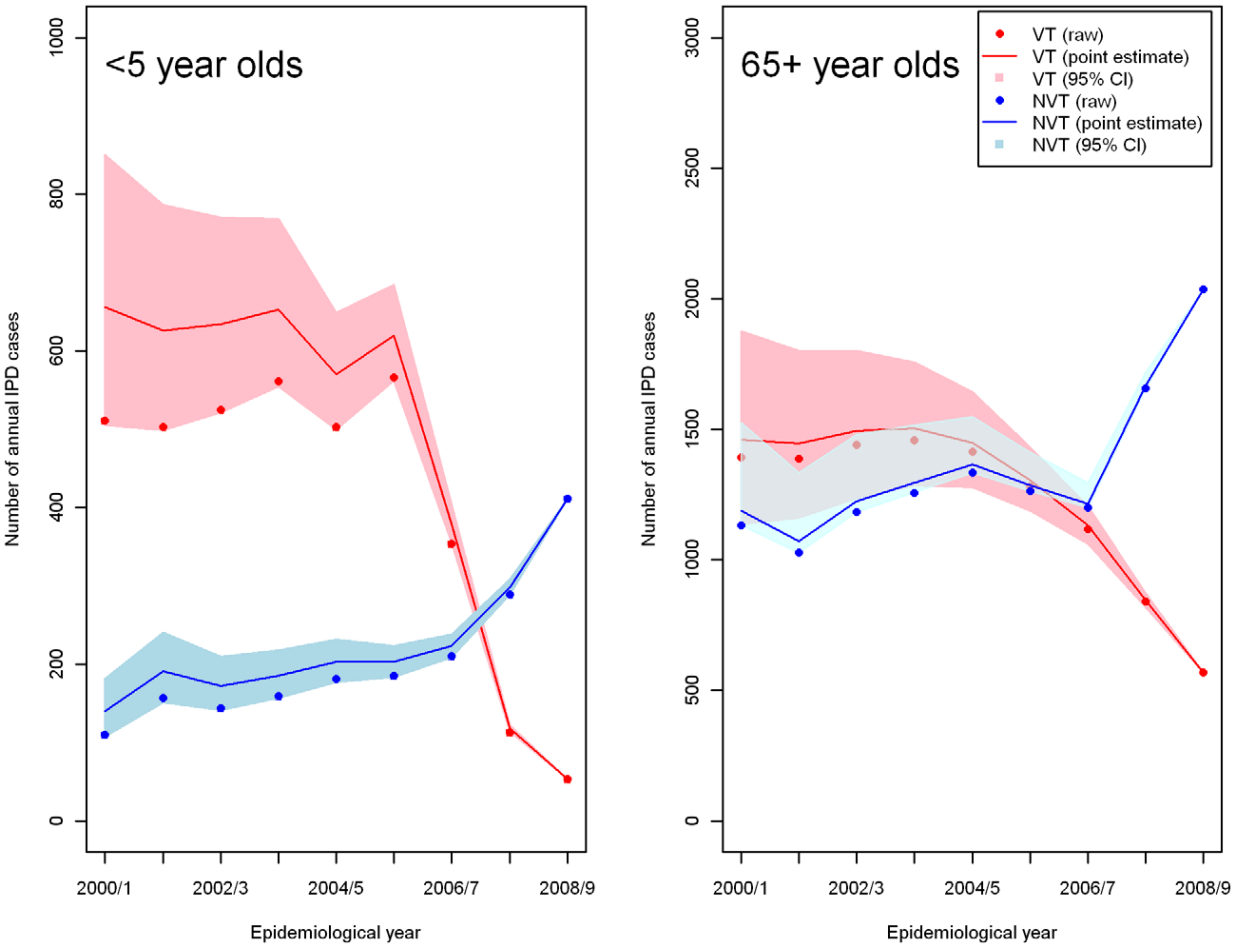
Raw (filled circles) annual PCV7 type (VT) and non-PCV7 type (NVT) IPD cases for < 5 and 65+ year olds and adjusted IPD cases (point estimate (solid lines) and 95% confidence intervals (shaded area)) by epidemiological year after correcting for the underlying upward trend in reporting in each age group.

The number of reports of IPD to the Health Protection Agency in the epidemiological year (the beginning of July to the end of June) before and for three epidemiological years since PCV7 introduction in England and Wales (up to the end of June 2009) was used for model fitting. Serotype 1 was excluded as it has shown large secular changes in incidence in the UK that are unrelated to PCV7 introduction [6]. Its transmission dynamics were therefore considered to be affected by factors other than those represented in the model. In the IPD data set, some cases were without information on age or serotype. Cases without age information in the dataset were distributed on the basis of the annual age distribution of cases with known ages. Then, cases without serotype information were distributed according to the annual serotype distribution of typed cases. There was an upward trend in overall IPD incidence from 2000/01 to 2005/06 before PCV7 introduction, paralleled by a similar upward trend in national reports of other bacteraemias [10]. These upward trends for other bacteraemias have continued post-PCV7 introduction and are likely to reflect improved ascertainment [6]. Based on these raw IPD data, an assumption was made that the upward trend in ascertainment of IPD cases in the pre-PCV7 period would have continued post-PCV. Age specific IPD rates were therefore adjusted by estimating the pre-vaccination trend *t* in total IPD by linear regression on logged titres (e.g *t* = 1.1 fold change per year) and inflating the raw incidence rates by a factor of *t* ^x^ where x is the number of epidemiological years prior to 2008/09. To obtain 95% CIs for the corrected rates the lower and upper 95% CI for *t* was used where the *t* values were estimated for six age groups: <2, 2–4, 5–14, 15–44, 45–64, and 65+. The adjusted IPD cases for two age groups (< 5 and 65+) are presented in Figure 2 (The full details of the ascertainment are available in Tables S1 and S2). Since these data are crucial to model predictions yet subject to an inherently uncertain ascertainment correction, three scenarios for the corrected rates were used, based on the point estimate, lower and upper limits of the 95% CIs.

In the pre-PCV7 use period, VT IPD accounted for 52% of the total IPD cases (75% for <5 and 49% for 5+ years). In contrast, in the third year of PCV7 use, the percentage of total IPD cases caused by VT was reduced to 20% (11% for <5 and 21% for 5+ years).

There is no cross protection from the 19F component against 19A as clearly evidenced by the increases in 19A seen in vaccinated children and older age groups in the UK and elsewhere. For 6C there is no evidence of cross protection from the 6B component of PCV7, and for 6A, while there is evidence for cross protection in vaccinated children against IPD, there is no evidence of herd immunity induced by cross protection as 6A cases have increased in older age groups in the UK [6]. We do not consider that cross protection within serogroups for PCV7 is an important factor to incorporate in the model.

#### PCV7 coverage

**Figure 3:**
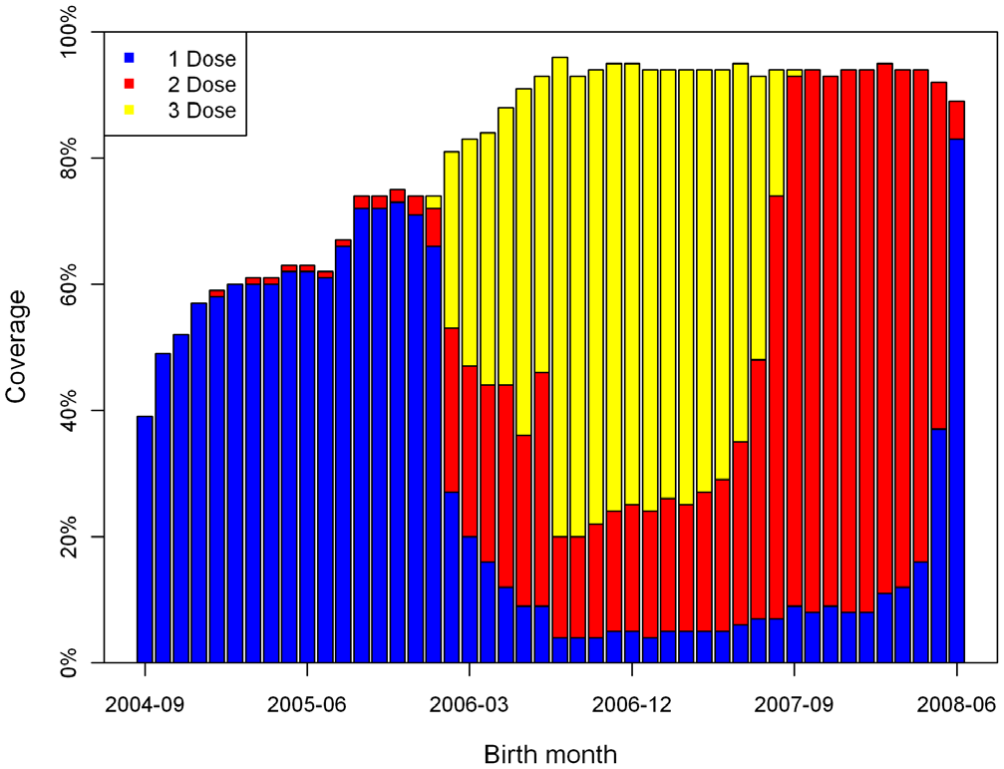
Cumulative PCV7 uptake by a number of doses for each birth-monthly age cohort at the end of September 2008 obtained from the General Practice Research Database.

The numbers of children receiving 0 to 3 doses of PCV7 by month for each monthly age cohort during the first two years of the PCV7 programme (October 2006 – September 2008) were obtained from the General Practice Research Database between October 2006 and September 2008. The first monthly birth cohort eligible for the routine PCV7 vaccination programme were children born in July 2006. Children older than this birth cohort but under two years as at September 2006 were scheduled for the catch-up campaign. Figure 3 presents the accumulated PCV7 uptake by three doses at the end of the two years period.

### Fitting procedure

The model was fitted to changes in pre- and post-PCV7 pneumococcal disease and carriage for both VT and NVT serotypes, by varying parameters representing vaccine efficacy against carriage and disease (for partially and fully protected individuals), the competition parameter measuring the protection against acquisition of NVT carriage for someone carrying VT, and the risk of transmission when a susceptible individual in the population has a physical contact with an infected individual [4]. Fitting was conducted by comparing (i) model estimates of pneumococcal carriage at pre-PCV7 equilibrium levels with data on pneumococcal carriage from a longitudinal swab study carried out in 2001/2, and (ii) reported IPD cases in the three years since the introduction of PCV7 (2006/7 to 2008/9). In order to generate predictions of IPD cases post-vaccination for the second comparison, case: carrier ratios were estimated for 16 age groups (<2 m, 2–3 m, 4–5 m, 6–11 m, 1, 2, 3, 4, 5–9, 10–14, 15–24, 25–44, 45–64, 65–74, 75–84, 85+.), with the prevalence of disease determined using pre-vaccination data. We assume that the risk of disease occurs at the time of carriage acquisition [5].

Optimal values of the parameters were obtained by minimising the Poisson deviance between model estimates and data. A simplex algorithm [7] (using the fminsearch routine in MATLAB R2006a) was used to find these parameters. This generated a range of best fitting models with different numbers of parameters governing the age-dependent risk of transmission when a susceptible individual contacts an infected individual. The best fitting of these models was chosen using Akaike's Information Criterion (AIC).

An alternative to the directed search approach such as the simplex algorithm we have used is to use a random search or Bayesian sampling approach to parameter estimation. This would produce a range of potential good fitting parameters rather than a single best fitting set, representing the uncertainty in the data being fitted to. However, we have taken this uncertainty into account to some extent, by conducting three separate fits to the midpoint and endpoints of the 95% uncertainty intervals in the annual number of IPD cases after adjusting for the pre-vaccination trend.

The model was then used to predict the long term effect of PCV7 introduction in England and Wales for fifty years after vaccination begins.

### Duration of carriage

As in the previous model, the average duration of carriage was estimated for the following age classes: 0–1, 2–4, 5–17 and 18+ [14], [15] and ranged from 72 days in the 0–1 year old age group to 17 days in the 18+ age group. Age-dependent recovery rates (1/average duration of carriage) were assumed to be the same for VT and NVT (rVi = rNi) due to no significant difference between these groups [14], [16], and also assumed to have the same rates for vaccinated and unvaccinated individuals. An exponential decay function of the recovery rates using these durations of infection has been formulated to provide age-dependent weekly recovery rates.

## Results

### Best fitting parameters

By fitting models to data, estimates of parameters governing vaccine efficacy, competition and risk of transmission per contact were obtained. The most parsimonious model (with the lowest AIC value) was that with six different age-dependent risks for transmission per contact, and hence this model was used.

The best fitting parameter for vaccine efficacy against disease (VEd) was close to 100% for both partially and fully protected groups (implying that partially and fully vaccinated carriers do not develop invasive disease). Vaccine efficacy against carriage in partially protected individuals was insensitive during the fitting process and was set to 50% of that in fully protected individuals (reported in Table 1). Hence there was only a single vaccine parameter (efficacy against carriage in the fully protected group or VEc) that influences goodness of fit. The best fitting value of the duration of vaccine protection was strongly correlated with the best fitting value of VEc; hence it was set to 8.3 years based on the PCV7 experience in the US [4] whilst the VEc was estimated in this study. The sensitivity analysis using the lower and upper boundaries of the duration of PCV7 protection (5 and 20 years) estimated from Melegaro et al. confirmed that there is only small difference between overall IPD cases after 10 years of the vaccine introduction resulted from three different durations (5, 8.3 and 20 years) of PCV7 protection but the fitting results estimated the degree of vaccine protection differently as the shorter duration of PCV protection the higher degree.

**Table 1:** Model deviance and best fitting values for the competition parameter (cN) and vaccine efficacy against carriage (VEc) with their 95% CIs based on three scenarios for the completeness of IPD ascertainment (best-fitting or point estimate, lower 95% limit of confidence interval and upper 95% limit of confidence interval).

Adjusted Scenarios	Deviance	VEc	95% CIs	c_N_	95% CIs
Point estimate	711.183	0.52	(0.51,0.56)	0.04	(0,0.18)
Lower 95% CI	709.202	0.51	(0.51,0.52)	0	(0,0.04)
Upper 95% CI	778.631	0.6	(0.56,0.65)	0.37	(0.24,0.47)

The best fitting competition parameter from the adjusted IPD data when the point estimate of the completeness of IPD ascertainment is used is 0.04 (Table 1) implying that the individuals with VT carriage have 96% (95% CI 82%-100%) protection against acquiring NVT carriage compared to susceptibles.

**Figure 4:**
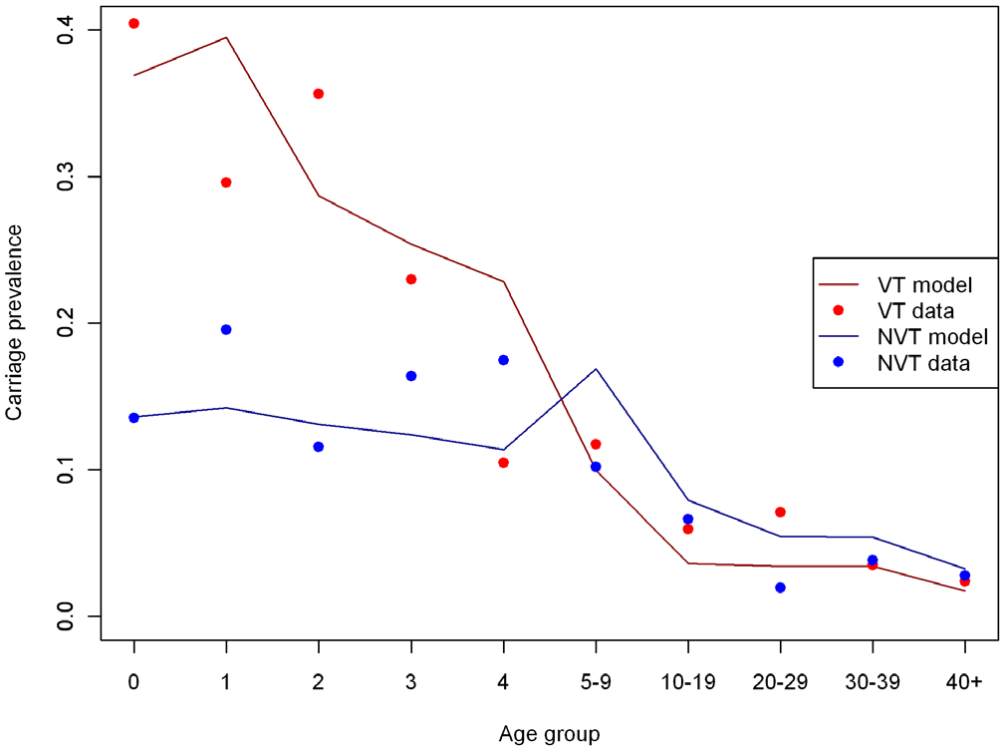
Carriage prevalence reported in the 2001/02 longitudinal swab study and best fitting model fitted values, stratified by serotype (VT or NVT) and 10 age groups. The model results represent the model fitted to the point estimate for the number of adjusted IPD cases, with serotype 1 excluded. The best fitting value for the competition parameter (0.04) is assumed here.

Results presented in Figure 4 show the carriage prevalence data of VT and NVT among 10 age groups and their best fitted values from the fitting model.

Using all contacts (rather than just physical contacts) made little difference. The best fitting probability of transmission in order to fit post-PCV-7PCV7 decline in IPD cases decreased because more contacts per individual were assumed. There was a marginally smaller reduction in overall IPD cases (41 accumulated cases during 10 years of PCV-7PCV7 use) from the long-term simulations using the mixing matrix with all contacts due to faster reduction in VT IPD cases but more replacement in NVT IPD cases.

### Case:carrier ratios

**Figure 5:**
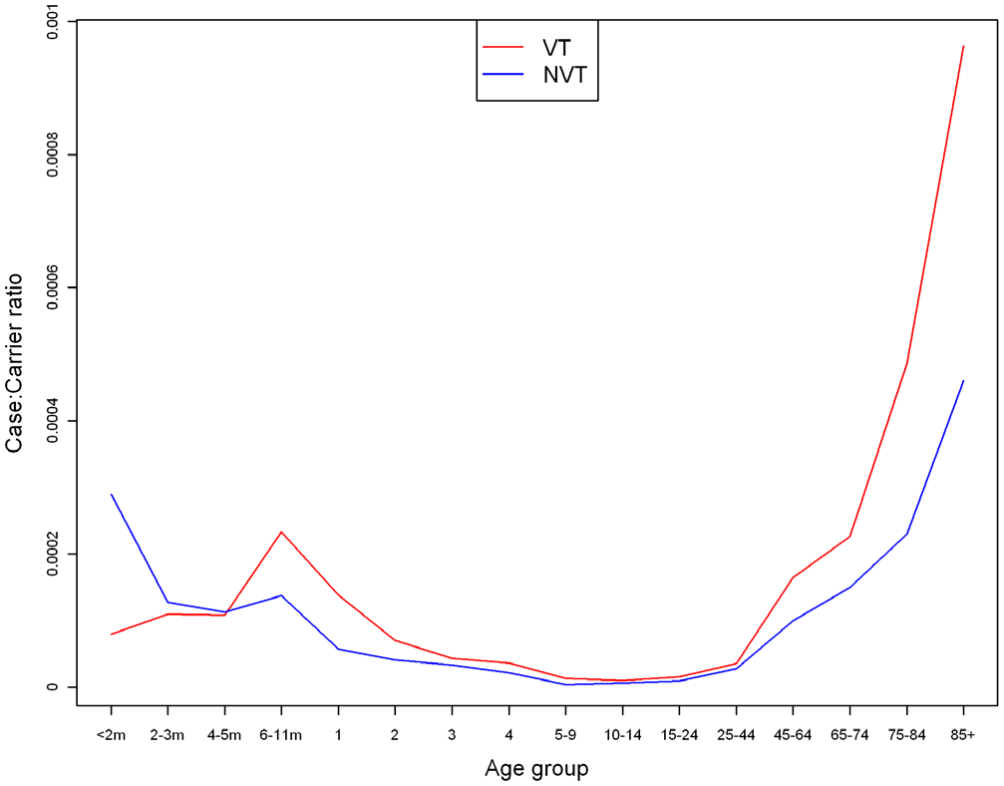
Case:carrier ratios of VT and NVT serotypes in 16 age groups for the best fitting model. The model results represent the model fitted to the point estimate for the number of adjusted IPD cases, with serotype 1 excluded.

The best fitting case:carrier ratios (Figure 5) suggest that acquisition of VT carriage has a higher risk of causing disease compared to those acquired NVT carriage for individuals over 6 months of age. They also suggest that the risk of invasive disease following any pneumococcal carriage acquisition is lower in individuals aged between 5 and 44 years compared to those aged <5 and 45+.

### Long term incidence

The best fitting model was used to predict the number of IPD cases expected in England and Wales for the ten years following PCV7 introduction. Elimination of VT IPD was predicted to occur within ten years of PCV7 introduction in the UK.

Predicted effect of PCV7 introduction on the number of NVT IPD cases for ten years after PCV7 introduction. The model results represent the model fitted to the point estimate for the number of adjusted IPD cases, with serotype 1 excluded. The shaded region shows the range of results obtained when the adjusted number of IPD cases is varied over its 95% CI.

**Figure 6:**
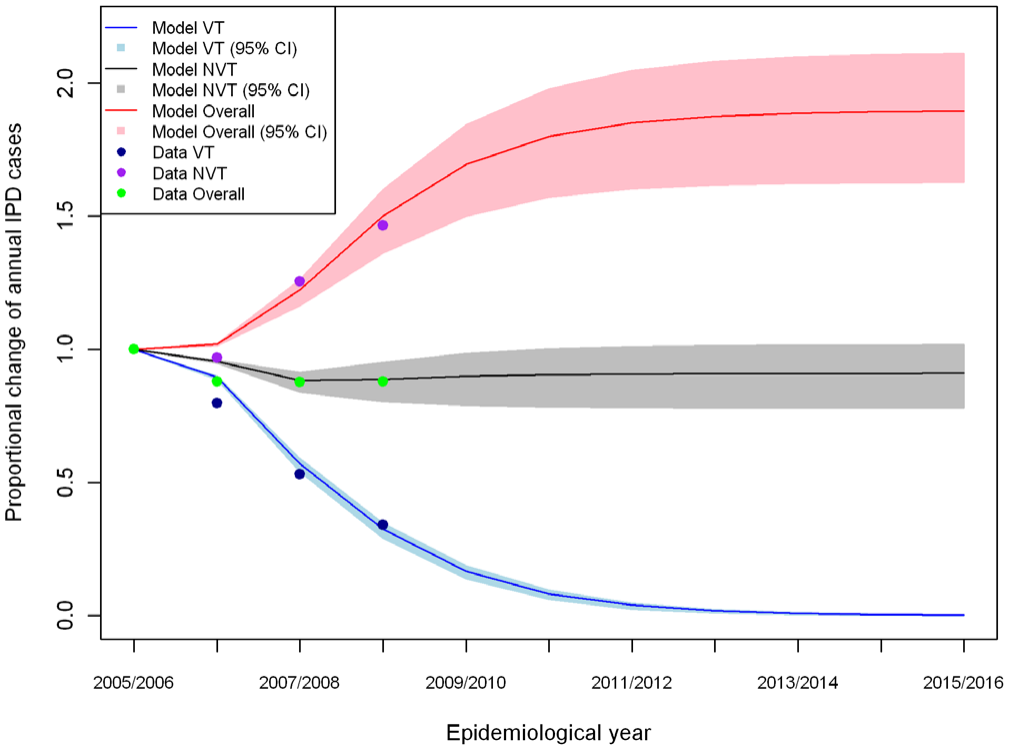
The proportional changes and their 95% CI areas of the number of overall and NVT IPD cases per year during the first ten years of PCV7 use in England and Wales predicted using the adjusted IPD cases with the best fitted ascertainment rate and its 95% CIs (Lines are for point estimates and shaded area for their 95% CIs).

The model suggests that the number of NVT IPD cases may increase by about 90% (95% CI 63% – 111%) after 10 years of PCV7 introduction (Figure 6). By the tenth year, the net change in the annual number of IPD cases (both VT and NVT) is estimated to be -9% (95% CI -22% - 2%) (Figure 6 and Table 2). The changes from the previous study are also included in Table 2 describing more reductions on the overall IPD cases due to less replacement in NVT IPD cases from the lower competition estimated using the US experience.

**Table 2:** Model estimated proportional change in NVT and overall number of IPD cases from pre- to 10 years post- PCV7 introduction in England and Wales, stratified by six age groups, based on the point estimate of the adjusted number of IPD cases, and 95% CIs represent the range of results obtained when the adjusted number of IPD cases is varied over its 95% CI.

	<5	5–14	15–44	45–64	65+	Total
NVT	136%	74%	87%	87%	87%	90%
95% CI	(84%,161%)	(55%,94%)	(62%,108%)	(62%,108%)	(62%,108%)	(63%,111%)
Melegaro et al. [5]	36%	24%	27%	28%	28%	28%
Overall	−42%	−26%	3%	−1%	−7%	−9%
95% CI	(−55%,−36%)	(−34%,−17%)	(−11%,15%)	(−14%,10%)	(−20%,3%)	(−22%,2%)
Melegaro et al. [5]	−63%	−31%	−21%	−28%	−42%	−38%

The proportional changes in NVT and overall IPD cases from best fitted model in the previous study [5] are included for comparison.

PCV7 is predicted to reduce the overall number of IPD cases by more in younger age groups (Table 2). This is due to both the direct effect of vaccination (administered to infants, and subject to waning in older age groups), as well as the higher level of serotype replacement in older adults as a result of the different distribution of PCV7 and non-PCV7 serotypes in different age groups. For instance, the proportion of IPD caused by PCV7 serotypes prior to PCV7 introduction was 75% in children aged <5 but only 50% in 65+ year olds.

The best fitting model predicted that there would be 5,700 (95% CIs, 700-13,000) fewer IPD cases over the first 10 years of vaccination compared to if PCV7 had not been introduced. However, if the number of IPD cases is not adjusted for the likely effects of improving ascertainment, then the best fitting model would instead predict that PCV7 introduction will cause 1,300 more cases over the same period.

## Discussion

Mathematical models have been widely used to investigate the impact of mass vaccination programmes on the burden of disease, as well as the cost effectiveness of such interventions [17]–[21]. Transmission dynamic models of vaccination programmes can take account of herd immunity effects which are often a major determinant of the overall population impact of the programme on disease burden. Such models have been developed to investigate the dynamics of pneumococcal transmission and the likely effect on IPD of PCV7 introduction. However, their reliability is critically dependent on the model structure and assumptions, and the adequacy of the data used for parameter estimation and model fitting. The model described here simulates the long term impact of the PCV7 programme in England and Wales on VT and NVT IPD, utilising the best IPD, carriage, vaccine coverage and population mixing data currently available for this population.

Another limitation of our model is that we did not build natural immunity into the model structure explicitly due to lack of information about the natural immunity. However, the assumption of natural immunity was implicitly accounted for by age-dependent transmission probabilities and case: carrier ratios, which are consistent with a decreasing probability of acquiring infection in adults and higher chance of developing IPD in young children and the elderly population. This method may slightly overestimate the impact of vaccination (since the parameters are fixed, whereas natural immunity may decrease if vaccination reduces the force of infection in children); however, the existence of natural immunity is so uncertain that it would be equally difficult to incorporate such an effect.

Despite the added realism in model structure and parameterisation, there is necessarily a limit to how far a model such as this one can be used to simulate reality, given the substantial differences between individual serotypes in case:carrier ratios, the potential need to incorporate natural immunity in the transmission model, together with the need to reflect transition between different states of vaccine-induced immunity as children receive their sequential doses in the immunisation course. These limitations will be particularly exposed when using such models to investigate the likely impact on IPD of higher valency vaccines such as the 10-valent (additionally covering serotypes 1, 5 and 7F) and the 13-valent (additionally covering serotypes 3, 6A and 19A). However, a model with more compartments representing individual serotypes and states of immunity would reach the limit of computational capacity within a compartmental model framework. Transition to an individual based modelling approach would seem to be the next logical step in pneumococcal model development.

Furthermore, the exclusion of serotype 1 from the model was necessitated by its idiosyncratic behaviour suggesting that the major determinants of its incidence were not those incorporated in our model. Secular changes in the incidence of certain serotypes have been noted in the past [22] and may be influenced by natural changes in population immunity (possibly induced by carriage) or possibly changes in the organism that affect its inherent transmissibility. However, serotype 1 is an important cause of IPD in adults aged between 5 and 64 years in the UK, and is a major pathogen in developing countries [23]. When estimating the likely benefit of higher valency conjugate vaccines that include serotype 1 it will therefore be important to include serotype 1 in the overall burden of potentially preventable VT IPD.

The results indicate that PCV7 will eliminate VT transmission in England and Wales within 10 years. Current VT incidence is consistent with that prediction [6]. Indeed, post-vaccination surveillance data from 2009/10 indicate an overall reduction in IPD of 56% in <2 year olds and 19% in ≥65 year olds, even better than the long-term reduction predicted by the model due to the recent reduction in IPD caused by serotype 1 (not included in the model). However, an increase in NVT IPD cases due to serotype replacement is inevitable. The magnitude of this effect is dependent on the competition parameter, c_N_, which reflects the protection that VT carriers have against acquiring NVT carriage. Based on the US post PCV7 data, VT carriers were estimated to have only 15% protection against acquiring NVT infection suggesting limited serotype replacement in the long term [5]. When using the UK post-PCV7 IPD data, VT carriers were estimated to have 96% (95% CIs, 72% – 100%) protection against acquiring NVT infections when compared to those who are susceptible to VT infections. This strong protection parameter causes a large replacement of VT by NVT IPD cases (Table 2). The long-term simulation model suggested PCV7 results in an overall change in IPD of −9% (95% CIs, −22% - 2%) by 10 years, substantially less than predicted from the previous model based on the US post PCV7 experience, and with some scenarios indicating an overall increase in IPD cases. However, the introduction of PCV13 in 2010 to the UK immunisation schedule has the potential to limit the increase in non-PCV7 serotypes.

One potential reason for the difference between c_N_ values estimated from the US and UK post PCV7 data may be the differences in IPD surveillance systems in two countries [5]. Unlike the national IPD data set in England and Wales, the US data from the Active Bacterial Core surveillance (ABCs) includes a substantial proportion of mild non-hospitalised paediatric cases who show no evidence of an increase in NVT IPD incidence [4]. When based on hospitalised cases however, the US experience appears more similar to that in England and Wales in terms of the absolute incidence and magnitude of increase in NVT IPD. It would therefore be of interest to parameterise a transmission dynamic model such as this one using post-PCV7 changes in VT and NVT IPD in the US just based on hospitalised cases. The reason for the lack of an increase in the incidence of NVT among non-hospitalised children in the ABCs system may be a reduction in the propensity to take a blood culture from those presenting with perhaps just a fever in the post-PCV7 era.

Several structures have been used in deterministic models in order to describe pneumococcal transmission and serotype interaction. Our model uses a “diamond” structure with four infected states (not infected, carrying VT infection, carrying NVT infection and co-infected with VT and NVT). Alternative model structures have been proposed which have the advantage of being “neutral null models” [24], i.e. having no intrinsic mechanism to promote stable co-existence of strains that are functionally indistinguishable. Two such models are a triangle model [24], [25](which does not allow co-infection of VT and NVT strains) and a pyramid model (which allows double infections by the same serotype group, and clearing of existing infection by a new infection when carrying double infections) [24]. The triangle model is unrealistic because many recent longitudinal nasopharyngeal studies with non-conventional methods for serotyping suggest frequent presence of multiple serotypes in individuals [26], [27]. A pyramid model is very attractive on theoretical grounds, but non-differentiated carriage data in terms of double infections from the same serotypes would be a major obstacle to estimating the parameters such a model for measuring effects of PCV introduction. We acknowledge that the structure chosen here has some less attractive features on theoretical grounds (i.e. it is non-neutral). However, it was chosen on a pragmatic basis, as it allows a reasonable estimate of the effects of PCV introduction starting from a non-zero baseline, within the limitations imposed to the available data.

Apart from the use of the pre- and post-PCV7 IPD data from England and Wales, there were other differences from the previous model [5] that were designed to represent more realistically the demographic and immunisation programmes in England and Wales. These included the addition of a partially protected group comprising of children under one year of age in receipt of a single dose, use of the actual age-specific coverage achieved in the routine and catch-up programmes, use of data on mixing patterns from the POLYMOD study rather than being estimated from the model, and a population structure based on the actual population of England and Wales. The mixing pattern estimated from the previous model parameterised with the US IPD data suggested very strongly assortative mixing (i.e. individuals have contacts mainly with other individuals in the same age group) while the POLYMOD data suggests more random mixing which leads to a greater herd immunity effect. For the population structure, the classical approaches are either to assume a uniform distribution with the same size age cohorts (generally from 0-69 years) or a distribution that uses the natural mortality rate in which the size of the age cohorts reduces exponentially (Figure S2). Both methods can result in unrealistic weights for specific age cohorts when calculating forces of infection, and may cause potential problems in estimating herd immunity. We used the real UK population structure in order to minimise these problems, although no allowance can be made for any future changes in population structure. These various improvements over the previous model should produce more accurate model predictions of the short term effects of the PCV7 programme (eg within the first few years) but will lead to similar longer-term end points due to the elimination of the VT carriage which is predicted to occur by year 10 of the PCV7 programme.

A model such as this that realistically represents herd immunity, replacement, population age structure and vaccine protection from both complete and incomplete schedules is important not just for England and Wales, but also for investigating vaccine impact in low and middle income settings. Such settings are likely to have heterogeneous vaccine schedules and fast changing population structures, with rapid growth in the size of younger age cohorts in particular. Indeed, an adapted version of this model is currently being used to inform work commissioned by the World Health Organization on optimising immunization schedules in low and middle income countries [28]. The current model, while yet to be parameterised for such countries, suggests that PCV7 vaccination can result in a substantial decrease in overall invasive pneumococcal disease in children even in an environment of rapid replacement with non-PCV7 serotypes. This underlines the importance of initiatives such as the Global Action Plan to combat childhood pneumonia with the use of tools such as pneumococcal conjugate vaccines. Our model can provide essential information for refining estimates of the cost-effectiveness of pneumococcal conjugate vaccine introduction, which are currently largely based on static models that may not accurately capture the dynamics of herd immunity and serotype replacement.

## Supporting Information

Figure S1 Dynamic model structure in equations.
(TIF)

Figure S2 Three methods of representing the age distribution of the population of England and Wales in mid-2005 (the total size of the population is the same in all three methods). (1-blue line. Actual population of England and Wales obtained from the Office for National Statistics, 2-red line. Rectangular age distribution assuming equally sized annual age cohorts up to 70 year olds, and 3-green line. Population subject to an age-dependent mortality rate.)
(TIF)

Table S1 Unadjusted and adjusted (95% confidence intervals) PCV7 type IPD incidence per 100,000 between 2000/01 and 2008/09 for six age groups with t values.
(DOC)

Table S2 Unadjusted and adjusted (with their 95% confidence intervals) non-PCV7 type IPD incidence per 100,000 between 2000/01 and 2008/09 for six age groups with t values. IPD cases due to serotype 1 were excluded.
(DOC)
