# Supplementary figures and images for: 7-Valent Pneumococcal Conjugate Vaccination in England and Wales: Is It Still Beneficial Despite High Levels of Serotype Replacement?
Source: PLoS One. 2011 Oct 14;6(10):e26190. doi: 10.1371/journal.pone.0026190 (PMC3193519; doi:10.1371/journal.pone.0026190)

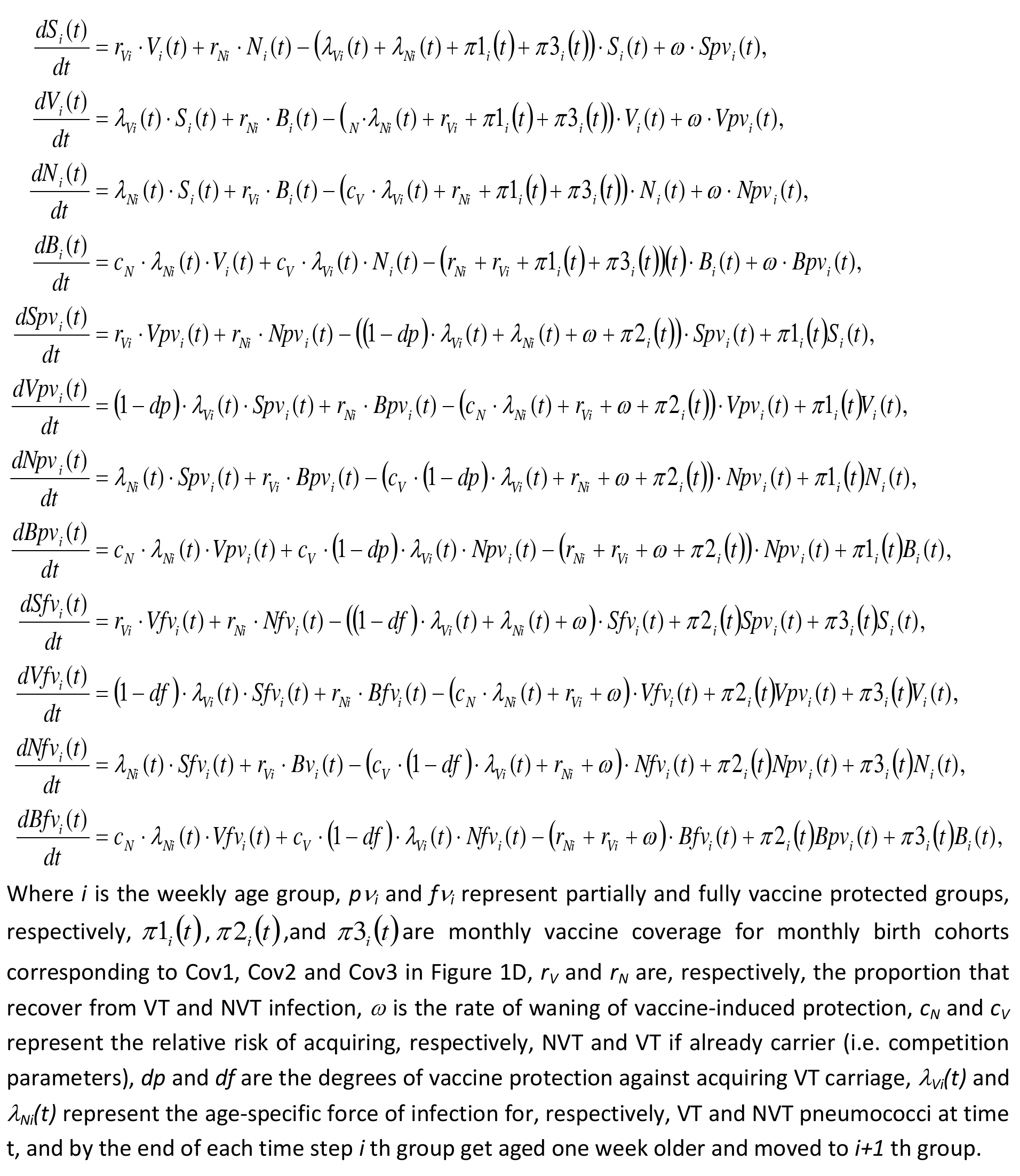

Supplement: Figure S1 — Dynamic model structure in equations. (TIF) [file pone.0026190.s001.tif]

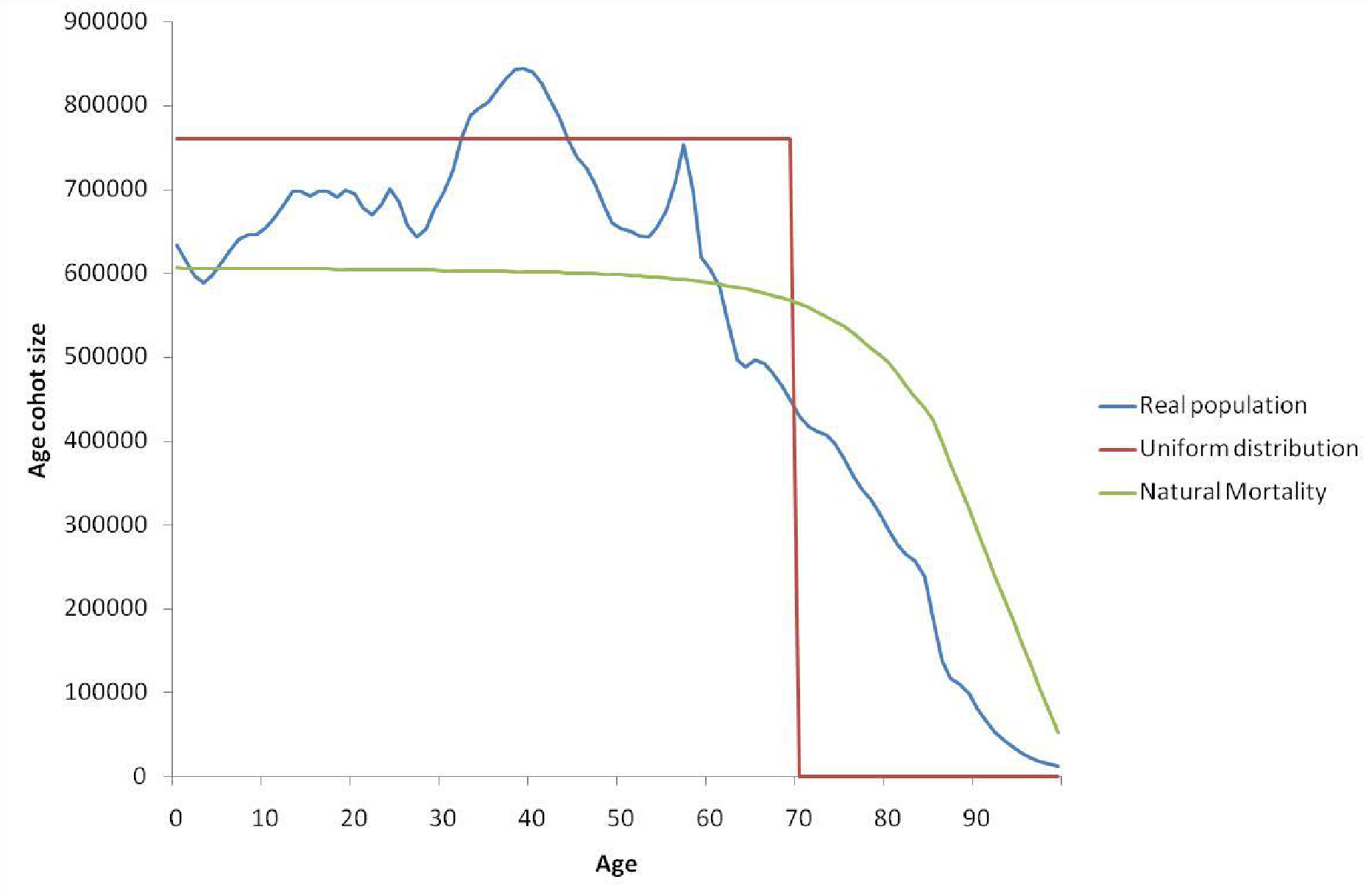

Supplement: Figure S2 — Three methods of representing the age distribution of the population of England and Wales in mid-2005 (the total size of the population is the same in all three methods). (1-blue line. Actual population of England and Wales obtained from the Office for National Statistics, 2-red line. Rectangular age distribution assuming equally sized annual age cohorts up to 70 year olds, and 3-green line. Population subject to an age-dependent mortality rate.) (TIF) [file pone.0026190.s002.tif]
