# Supplementary material for: 7-Valent Pneumococcal Conjugate Vaccination in England and Wales: Is It Still Beneficial Despite High Levels of Serotype Replacement?
Source: PLoS One. 2011 Oct 14;6(10):e26190. doi: 10.1371/journal.pone.0026190 (PMC3193519; doi:10.1371/journal.pone.0026190)
Supplement: Table S1 — Unadjusted and adjusted (95% confidence intervals) PCV7 type IPD incidence per 100,000 between 2000/01 and 2008/09 for six age groups with t values. (DOC) [file pone.0026190.s003.doc]

| **age group** | **data** | **2000/01** | **2001/02** | **2002/03** | **2003/04** | **2004/05** | **2005/06** | **2006/07** | **2007/08** | **2008/09** |
| --- | --- | --- | --- | --- | --- | --- | --- | --- | --- | --- |
| **<2** | Unadjusted | 31.13 | 31.29 | 32.88 | 35.86 | 30.16 | 33.45 | 17.64 | 3.97 | 2.41 |
|  | Adjusted (1.02) | 37.34 | 36.69 | 37.69 | 40.18 | 33.04 | 35.81 | 18.46 | 4.06 | 2.41 |
|  | 95% CI (0.99,1.06) | (28.49,48.87) | (28.96,46.43) | (30.77,46.11) | (33.93,47.54) | (28.86,37.80) | (32.36,39.61) | (17.25,19.74) | (3.92,4.20) |  |
|  |  |  |  |  |  |  |  |  |  |  |
| **2-4** | Unadjusted | 6.68 | 6.83 | 7.43 | 7.28 | 7.32 | 7.93 | 6.93 | 3.24 | 1.03 |
|  | Adjusted (1.06) | 10.25 | 9.94 | 10.24 | 9.52 | 9.07 | 9.32 | 7.72 | 3.41 | 1.03 |
|  | 95% CI (1.02,1.09) | (8.08,13.02) | (8.06,12.25) | (8.56,12.25) | (8.20,11.05) | (8.05,10.22) | (8.52,10.19) | (7.27,8.19) | (3.31,3.52) |  |
|  |  |  |  |  |  |  |  |  |  |  |
| **5-14** | Unadjusted | 0.77 | 0.63 | 0.7 | 0.84 | 1.11 | 0.99 | 0.85 | 0.65 | 0.46 |
|  | Adjusted (1.08) | 1.43 | 1.08 | 1.12 | 1.24 | 1.52 | 1.25 | 1 | 0.7 | 0.46 |
|  | 95% CI (1.02,1.15) | (.91,2.26) | (.73,1.62) | (.80,1.58) | (.93,1.66) | (1.21,1.91) | (1.05,1.48) | (.89,1.12) | (.66,.74) |  |
|  |  |  |  |  |  |  |  |  |  |  |
| **15-44** | Unadjusted | 1.43 | 1.32 | 1.5 | 1.74 | 1.78 | 1.58 | 1.56 | 1.05 | 0.7 |
|  | Adjusted (1.06) | 2.73 | 2.02 | 2.15 | 2.34 | 2.27 | 1.89 | 1.76 | 1.12 | 0.7 |
|  | 95% CI (1,1.13) | (1.45,3.79) | (1.33,3.10) | (1.51,3.11) | (1.74,3.18) | (1.79,2.90) | (1.58,2.27) | (1.56,1.99) | (1.05,1.19) |  |
|  |  |  |  |  |  |  |  |  |  |  |
| **45-64** | Unadjusted | 3.26 | 3.15 | 3.81 | 3.96 | 4.08 | 4.74 | 3.83 | 3.08 | 2.12 |
|  | Adjusted (1.09) | 6.35 | 5.65 | 6.29 | 6.01 | 5.7 | 6.09 | 4.53 | 3.35 | 2.12 |
|  | 95% CI (1.07,1.11) | (5.55,7.24) | (5.03,6.34) | (5.69,6.94) | (5.53,6.53) | (5.33,6.09) | (5.79,6.40) | (4.38,4.68) | (3.29,3.40) |  |
|  |  |  |  |  |  |  |  |  |  |  |
| **65+** | Unadjusted | 16.8 | 16.63 | 17.15 | 17.25 | 16.62 | 14.94 | 12.98 | 9.67 | 6.43 |
|  | Adjusted (1) | 17.62 | 17.34 | 17.78 | 17.77 | 17.02 | 15.21 | 13.13 | 9.73 | 6.43 |
|  | 95% CI (0.98,1.04) | (13.72,22.64) | (13.93,21.59) | (14.73,21.45) | (15.20,20.79) | (15.02,19.29) | (13.85,16.71) | (12.34,13.98) | (9.43,10.04) |  |
