# Supplementary material for: 7-Valent Pneumococcal Conjugate Vaccination in England and Wales: Is It Still Beneficial Despite High Levels of Serotype Replacement?
Source: PLoS One. 2011 Oct 14;6(10):e26190. doi: 10.1371/journal.pone.0026190 (PMC3193519; doi:10.1371/journal.pone.0026190)
Supplement: Table S2 — Unadjusted and adjusted (with their 95% confidence intervals) non-PCV7 type IPD incidence per 100,000 between 2000/01 and 2008/09 for six age groups with t values. IPD cases due to serotype 1 were excluded. (DOC) [file pone.0026190.s004.doc]

| **age group** | **data (*t*)** | **2000/01** | **2001/02** | **2002/03** | **2003/04** | **2004/05** | **2005/06** | **2006/07** | **2007/08** | **2008/09** |
| --- | --- | --- | --- | --- | --- | --- | --- | --- | --- | --- |
| **<2** | Unadjusted | 7.18 | 11.21 | 9.67 | 10.35 | 12.16 | 10.87 | 13.09 | 16.42 | 21.22 |
|  | Adjusted (1.02) | 8.62 | 13.14 | 11.08 | 11.6 | 13.32 | 11.64 | 13.7 | 16.8 | 21.22 |
|  | 95% CI (0.99,1.06) | (6.57,11.28) | (10.38,16.63) | (9.05,13.56) | (9.80,13.73) | (11.63,15.23) | (10.52,12.87) | (12.80,14.65) | (16.24,17.38) |  |
|  |  |  |  |  |  |  |  |  |  |  |
| **2-4** | Unadjusted | 1.14 | 1.22 | 1.62 | 1.96 | 1.72 | 2.64 | 2.26 | 3.81 | 6.19 |
|  | Adjusted (1.06) | 1.76 | 1.78 | 2.23 | 2.56 | 2.14 | 3.09 | 2.52 | 4.02 | 6.19 |
|  | 95% CI (1.02,1.09) | (1.38,2.23) | (1.44,2.19) | (1.86,2.67) | (2.20,2.97) | (1.90,2.41) | (2.83,3.38) | (2.37,2.67) | (3.90,4.14) |  |
|  |  |  |  |  |  |  |  |  |  |  |
| **5-14** | Unadjusted | 0.44 | 0.5 | 0.77 | 0.48 | 0.5 | 0.73 | 0.82 | 1.09 | 1.05 |
|  | Adjusted (1.08) | 0.83 | 0.86 | 1.23 | 0.71 | 0.69 | 0.93 | 0.96 | 1.18 | 1.05 |
|  | 95% CI (1.02,1.15) | (.53,1.31) | (.58,1.29) | (.87,1.73) | (.54,.95) | (.55,.87) | (.78,1.10) | (.86,1.08) | (1.11,1.25) |  |
|  |  |  |  |  |  |  |  |  |  |  |
| **15-44** | Unadjusted | 1.55 | 1.4 | 1.61 | 1.83 | 2.22 | 1.95 | 2.11 | 2.88 | 3.15 |
|  | Adjusted (1.06) | 2.93 | 2.13 | 2.32 | 2.47 | 2.82 | 2.34 | 2.38 | 3.06 | 3.15 |
|  | 95% CI (1,1.13) | (1.56,4.09) | (1.41,3.26) | (1.62,3.34) | (1.84,3.35) | (2.23,3.60) | (1.96,2.81) | (2.11,2.69) | (2.88,3.25) |  |
|  |  |  |  |  |  |  |  |  |  |  |
| **45-64** | Unadjusted | 3.51 | 3.74 | 4.07 | 4.47 | 5.04 | 5.36 | 5.35 | 6.82 | 8.23 |
|  | Adjusted (1.09) | 6.84 | 6.71 | 6.71 | 6.78 | 7.04 | 6.89 | 6.32 | 7.41 | 8.23 |
|  | 95% CI (1.07,1.11) | (5.98,7.80) | (5.97,7.53) | (6.07,7.40) | (6.23,7.36) | (6.59,7.52) | (6.55,7.24) | (6.11,6.53) | (7.29,7.53) |  |
|  |  |  |  |  |  |  |  |  |  |  |
| **65+** | Unadjusted | 13.67 | 12.31 | 14.08 | 14.87 | 15.66 | 14.73 | 13.95 | 19.05 | 23.08 |
|  | Adjusted (1) | 14.34 | 12.84 | 14.59 | 15.32 | 16.04 | 15 | 14.11 | 19.17 | 23.08 |
|  | 95% CI (0.98,1.04) | (11.16,18.42) | (10.31,15.98) | (12.09,17.61) | (13.10,17.92) | (14.16,18.18) | (13.65,16.48) | (13.26,15.03) | (18.58,19.78) |  |
